# Enhanced Immunogenicity of Inactivated Dengue Vaccines by Novel Polysaccharide-Based Adjuvants in Mice

**DOI:** 10.3390/microorganisms10051034

**Published:** 2022-05-16

**Authors:** Shuenn-Jue Wu, Dan Ewing, Appavu K. Sundaram, Hua-Wei Chen, Zhaodong Liang, Ying Cheng, Vihasi Jani, Peifang Sun, Gregory D. Gromowski, Rafael A. De La Barrera, Megan A. Schilling, Nikolai Petrovsky, Kevin R. Porter, Maya Williams

**Affiliations:** 1 Viral and Rickettsial Diseases Department, Infectious Diseases Directorate, Naval Medical Research Center, Silver Spring, MD 20910, USA; daniel.f.ewing.civ@mail.mil (D.E.); appavu.k.sundaram.ctr@mail.mil (A.K.S.); huawei.chen2.ctr@mail.mil (H.-W.C.); zhaodong.liang.ctr@mail.mil (Z.L.); ying.cheng4.ctr@mail.mil (Y.C.); vihasi.s.jani.ctr@mail.mil (V.J.); peifang.sun2.civ@mail.mil (P.S.); megan.a.schilling.mil@mail.mil (M.A.S.); 2 Henry M. Jackson Foundation for the Advancement of Military Medicine, Bethesda, MD 20817, USA; 3 Leidos, Inc., Reston, VA 20190, USA; 4 Viral Diseases Branch, Walter Reed Army Institute of Research, Silver Spring, MD 20910, USA; gregory.d.gromowski.civ@mail.mil; 5 Pilot Bioproduction Facility, Walter Reed Army Institute of Research, Silver Spring, MD 20910, USA; rafael.a.delabarrera.ctr@mail.mil; 6 Vaxine Pty Ltd., Warradale, SA 5042, Australia; nikolai.petrovsky@flinders.edu.au; 7 College of Medicine and Public Health, Flinders University, Bedford Park, SA 5042, Australia; 8 Infectious Diseases Directorate, Naval Medical Research Center, Silver Spring, MD 20910, USA; kevin.r.porter3.civ@mail.mil (K.R.P.); maya.williams1.mil@mail.mil (M.W.)

**Keywords:** dengue virus, formalin inactivation, psoralen inactivation, dengue vaccine, polysaccharide-based adjuvants, Advax™ adjuvants

## Abstract

Dengue fever, caused by any of four dengue viruses (DENV1-4), is a major global burden. Currently, there is no effective vaccine that prevents infection in dengue naïve populations. We tested the ability of two novel adjuvants (Advax-PEI and Advax-2), using aluminum hydroxide (alum) as control, to enhance the immunogenicity of formalin- or psoralen-inactivated (PIV or PsIV) DENV2 vaccines in mice. Mice were vaccinated on days 0 and 30, and serum samples were collected on days 30, 60, 90, and 101. Neutralizing antibodies were determined by microneutralization (MN) assays, and the geometric mean 50% MN (MN_50_) titers were calculated. For the PIV groups, after one dose MN_50_ titers were higher in the novel adjuvant groups compared to the alum control, while MN_50_ titers were comparable between the adjuvant groups after the second dose. For the PsIV groups, both novel adjuvants induced higher MN_50_ titers than the alum control after the second dose. Spleen cells were collected on days 45 and 101 for enzyme-linked immunospot (ELISPOT) for IFNγ and IL4. Both PIV and PsIV groups elicited different degrees of IFNγ and IL4 responses. Overall, Advax-2 gave the best responses just ahead of Advax-PEI. Given Advax-2’s extensive human experience in other vaccine applications, it will be pursued for further development.

## 1. Introduction

Dengue fever is regarded globally as the most important arthropod-borne viral disease. Dengue virus infection can cause debilitating dengue fever and the more severe dengue fever, previously known as dengue hemorrhagic fever. The estimated population living in areas at risk for dengue infection is two billion people, and 390 million infections occur annually, primarily in tropical and subtropical climes [1]. Dengue fever, transmitted primarily by *Aedes* mosquitoes, is caused by any of the four serotypes of dengue virus (DENV1-4) and is one of the fastest growing global health concerns and a major infectious disease threat to U.S. military personnel deployed to endemic areas [2]. The importance of dengue to the U.S. military is highlighted by the fact that it is ranked third on the 2019 Global Ranked List of Infectious Disease Threats of U.S. Military Importance [3]. The development of an effective vaccine to prevent dengue fever is a priority for both the U.S. Department of Defense (DoD) and the World Health Organization (WHO). 

Despite nearly sixty years of sustained effort, an effective dengue vaccine for dengue naïve populations, including deployed U.S. military personnel, has still not been developed. This is primarily due to the need for balanced tetravalent immunity to all four dengue serotypes [4,5]. Dengue has distinct epidemiological patterns associated with four closely related serotypes of the virus. These serotypes can co-circulate within an endemic region, and thus many countries are hyper-endemic for all four serotypes [6]. As an RNA flavivirus, dengue virus population does not consist of a single genotype but is an ensemble of related mutants and recombinant viral genomes, named quasispecies. The high mutation rates of RNA viruses often cause the development of resistance to antiviral drugs and antibodies elicited by vaccines, making it very difficult for therapeutics and vaccines to work effectively [7]. The difficulty of DENV vaccine development is compounded by the presence of four serotypes and the phenomenon of Antibody-Dependent Enhancement (ADE) [4,5]. Each DENV serotype is sufficiently different, such that there is no cross-protection between serotypes (only transient cross-neutralizing antibodies), and epidemics caused by multiple serotypes (hyper-endemicity) can result. ADE occurs when antibodies from a primary infection bind but do not neutralize the virus during a secondary infection, and the virus-antibody complex gains entry into target cells via the Fc receptor, which predisposes an individual to an enhanced severity of disease upon re-infection with a different serotype [4,5]. Thus, it is essential that a successful DENV vaccine induce an adequate and equal level of protection against all four serotypes simultaneously to prevent ADE.

The first licensed dengue vaccine (Dengvaxia^®^) (Sanofi Pasteur, Lyon, France) was approved in Mexico on 9 December 2015 and is based on a live viral vector that needs to be administered three times over 12 months. Due to its less than desirable immunogenicity and efficacy, the U.S. Food and Drug Administration (FDA) has only approved this vaccine “for use in individuals 9–16 years of age with laboratory-confirmed previous dengue infection and living in endemic areas” (FDA letter 1 May 2019). In that letter, it also listed limitations of use such as “Dengvaxia^®^ is not approved for use in individuals not previously infected by any dengue virus serotype or for whom this information is unknown. Those not previously infected are at increased risk for severe dengue disease when vaccinated and subsequently infected with dengue virus.” Therefore, this vaccine is not suitable for U.S. military or general traveler populations. It is thereby imperative to continue exploring alternative dengue vaccine candidates, ideally based on inactivated platforms that do not suffer from the same variability and long lead times before protection is achieved as is seen with live vaccines [4,5]. Past experience with live attenuated viruses and the suboptimal results from Sanofi-Pasteur’s Dengvaxia^®^ vaccine clinical trials suggest that the next generation dengue vaccines will need to be based on non-replicating virus platforms such as inactivated viruses. In the case of live virus replicating vaccines, striking a balance between immunogenicity and attenuation of each DENV serotype, and attaining uniformity of immune responses to four serotypes in a mixed tetravalent formulation, have proven difficult. Purified inactivated viruses (PIVs) are attractive vaccine candidates because they are often safer than live virus vaccines and are generally stable and easy to maintain [4,5]. However, inactivated dengue virus vaccine candidates have shortcomings including short-lived antibody responses and a lack of cellular immune responses [4]. One way to potentially overcome some of these shortcomings is through the use of novel adjuvants that could enhance the magnitude and duration of the immune response to the inactivated vaccines. Select adjuvants may also be able to reduce any risk of ADE by reshaping the immune response to the virus.

Purified inactivated dengue vaccine candidates (PIV) have been developed using formalin for inactivation and have been tested using novel adjuvants and alum in more than 200 human subjects as well as a large number of non-human primates (NHPs) [8,9,10,11,12]. However, formalin inactivation is known to damage the antigenic structure of viruses. As a possible alternative, inactivated dengue vaccine candidates have also been developed using inactivation methods that work at the nucleic acid level (photo-inactivation in the presence of a psoralen compound) rather than protein level, thereby leaving the antigenic structure of the virus in its native form [13]. These psoralen-inactivated dengue vaccine (PsIV) candidates elicited high levels of virus neutralizing antibodies in vaccinated mice and NHPs [13,14]. In addition, PsIV-induced T-cell responses have been shown to be equivalent to a live dengue virus vaccine candidate [13]. 

The testing of adjuvants that may enhance the immunogenicity of inactivated dengue vaccine candidates is important to advance dengue vaccine efforts. In the selection of adjuvants that are suitable for a human vaccine, the vaccine maker needs to consider at least six major elements, such as intellectual property (public domain), efficacy, safety, ease of use, ease of manufacture, and cost [15]. Taking into consideration these elements, we identified two novel polysaccharide-based adjuvants to test, Advax-2 and Advax-PEI, developed by Vaxine Pty Ltd. (Adelaide, Australia). Advax™ is based on nanoparticles of delta inulin, which is a natural plant-derived polysaccharide. This polysaccharide-based Advax can be formulated with additional toll-like receptor (TLR) ligands to produce potent combination adjuvant formulations. Two Advax derivatives, Advax-2, containing a potent human and mouse TLR9 agonist CpG55.2, and Advax-PEI, with surface modification to induce antigen binding to the delta inulin surface, were used in this study. Advax-2 formulations have demonstrated faster induction of protective immunity, induction of broadly cross-neutralizing antibodies, and long-lasting memory CD4 and CD8 T-cell responses as well as a favorable safety profile in clinical trials of COVID-19, influenza, hepatitis B, and allergy vaccines involving over 20,000 human subjects to date and is contained in a now licensed COVID-19 vaccine [16,17,18]. Another potential benefit of Advax-2 is that it has been shown to prevent vaccine-enhanced disease in several different viral disease models, preventing SARS vaccine-associated eosinophilic pneumonitis in a SARS coronavirus disease model [19], and also preventing Japanese encephalitis (JE) vaccine antibody-dependent enhanced cellular infection by DEN1 and DEN2 in an in vitro dengue ADE model [20]. Notably, ADE was apparent in both cases when mice were immunized with the same antigens without Advax-2 or formulated with alum adjuvant, confirming that the prevention of ADE in both models was Advax-2 adjuvant dependent.

In this study, we tested these two novel adjuvants with either PIV or PsIV DENV2 monovalent vaccines in mice. We chose DENV2 for this proof-of-concept study due to availability of DENV2 PIV from our coauthors at WRAIR and DENV2 PsIV from our team at NMRC. Mice have traditionally been used as a first screen for DENV vaccine candidates. As mice are not an ideal model for dengue vaccines, we typically only use mice for the initial screening to down-select lead candidates for confirmatory testing in non-human primates (NHP). Therefore, this study used monovalent DENV2 to test the different adjuvants, although the plan will be to ultimately test a tetravalent vaccine in NHP. Due to the inability to quantitate the inactivated vaccines using the same units of measurement, this study was not designed to be an exact head-to-head comparison of the PIV and PsIV products but was meant to determine the best adjuvant for each vaccine candidate. The immunogenicity results for both B cell and T-cell responses presented here are intended to inform the down-selection of candidate vaccine-adjuvant combinations for ongoing development.

## 2. Materials and Methods

### 2.1. Chemical Inactivation of DENV2 Particles

#### 2.1.1. DENV2 Inactivation Using Formalin

DENV2 PIV (non-GMP Lot 1700; manufactured by WRAIR PBF; 18 µg/mL frozen stock) was obtained from our coauthors at WRAIR. DENV2 (strain S16803) was sucrose gradient purified and inactivated with formalin using a previously published procedure [8,21]. Briefly, DENV2 viral fluids were propagated in Vero cells, harvested, clarified, and purified using an established sucrose gradient methodology. The purified material was inactivated using formalin (pH 7.4) at a final concentration of 0.02%, for 10 days at 22 °C. Residual impurities, such as formalin, were removed from the final vaccine preparation using a tangential flow filtration column. 

#### 2.1.2. DENV2 Inactivation Using Psoralen

DENV2 PsIV has been previously prepared by our team and used in published mouse and NHP studies [12]. DENV2 (strain Philippines 2-012-84) was purified using Capto DeVirS chromatography resin and was then inactivated using a psoralen derivative 4′-aminomethyl-4,5′,8-trimethylpsoralen (AMT) (Cayman Chemical, Ann Arbor, MI, USA) following a previously published paper with minor modification [13]. Briefly, AMT was added to purified virus to a final concentration of 50 µg of AMT/mL of virus. The mixture was then exposed to UV irradiation (365 nm) at 500 µW/cm^2^, for 40 min. Psoralen inactivated DENV2 particles (DENV2 PsIV) were then further purified using Capto Core 700 column, as described previously [14]. DENV2 PsIV particles were collected in the void volume of the column (because molecules larger than 700 K molecular weight were excluded from entering the Capto Core 700 beads) and pooled together. The stabilizing agent FTA (1% recombinant human serum albumin, 15% trehalose, and 2% pluronic F-127) was added to DENV2 PsIV, which was then stored as 1 mL aliquots at −80 °C.

### 2.2. Quantitation of DENV2 PsIV and PIV

DENV2 PsIV particles were quantitated as particles/mL using Virocyt 3100 (Sartorius, Germany) according to the manufacturer’s protocol. DENV2 PsIV was prepared as the stock at 3.4 × 10^8^ particles/mL and then formulated as 10^5^ virus particles per dose with each adjuvant for evaluation in mice. WRAIR DENV2 PIV non-GMP Lot-1700 was used at 350 ng dose by diluting from the 18 µg/mL frozen stock. The aim was to select a suboptimal dose of each antigen based on recent studies [14,22] so the impact of the different adjuvants could be more easily seen. 

### 2.3. Preparation of Vaccine-Adjuvant Formulations

Advax-PEI and Advax-2 adjuvants were supplied as 50 mg/mL of suspensions of particles of delta inulin in an aqueous form (Vaxine). Advax adjuvants were administered at 1 mg/dose in mice. Advax-PEI was delta inulin alone, while Advax-2 was delta inulin plus CpG55.2 oligonucleotide. Alhydrogel^®^ adjuvant 2%, an aluminum hydroxide wet gel suspension (Invitrogen, Brenntag Biosector, Ballerup, Denmark) (referred to as alum), was added to the antigen at 1:9 ratio (alum:antigen) to prepare the alum adjuvanted vaccine doses. All vaccine-adjuvant formulations were prepared by gentle mixing immediately prior to each immunization.

### 2.4. Animals and Vaccine Administration

The animal protocol #19-IDD-10 for this mouse study was reviewed and approved by the WRAIR/NMRC Institutional Animal Care and Use Committee (IACUC) in compliance with all applicable federal regulations governing the protection of animals in research. Housing and experimental use of the animals were performed in strict accordance with all applicable federal regulations governing the protection of animals and research. Seventy BALB/c mice (6–8 weeks old, female) (Charles River Laboratories, Wilmington, MA, USA) were distributed into seven groups of 10 mice each. Groups of 10 mice were immunized with different vaccine-adjuvant formulations, as shown in Table 1. Mice were vaccinated with formalin-inactivated DENV2 (PIVD2) (350 ng per dose) or psoralen-inactivated DENV2 (PsIVD2) (10^5^ inactive virus particles per dose) mixed with adjuvant in a final volume of 50 µL intramuscularly on days 0 and 30 in alternate thighs. An adjuvant control group was injected with alum only. As shown in Table 2, five mice from each group were used for serum collections on days 0, 30, 60, 90, and 101 to monitor the longevity of neutralizing antibodies. The other five mice from each group were euthanized on day 45 for the collection of spleen cells to measure T-cell responses. The remaining five mice from each group were injected with a third dose of vaccine-adjuvant formulations on day 90 to enable measurement of T-cell responses 11 days after boosting, i.e., day 101, as T-cell responses usually peak approximately two weeks after immunization. These boosted mice were euthanized on day 101, and their spleens were harvested to measure T-cell responses.

### 2.5. Microneutralization Tests to Measure Neutralizing Antibody Responses

Individual serum samples from each group for each time point were tested for the presence of anti-DENV2 neutralizing antibodies using a high throughput microneutralization (MN) test with Vero cells, as previously described [23]. Briefly, two-fold serial dilutions of serum samples were incubated with two hundred Tissue Culture Infectious Dose (TCID5) of DENV2 in triplicate for 60 min in a 96-well flat bottom microtiter plate. Vero 81 cells (2 × 10^4^ per well) were then added to the microtiter plate and incubated at 37 °C for 4–5 days. Cells were fixed and DENV2-specific antigen was measured using rabbit anti-DENV polyclonal antibody and a peroxidase labeled anti-rabbit IgG secondary antibodies in an ELISA format. The highest reciprocal serum dilution that resulted in >50% reduction in absorbance compared to a virus control (lacking serum) was determined as the 50% MN titer (expressed as MN_50_ titers). The geometric mean MN_50_ titers were determined for each group for all time points. The geometric mean is used here because it deals with outliers better and is usually used with antibody titers. Seropositivity was defined as a titer ≥ 1:20. A one-way ANOVA with Dunnett’s post hoc test was performed using GraphPad Prism 9 to test for differences in neutralizing antibody titers between the alum group and the other adjuvant groups.

### 2.6. ELISPOT Assay to Measure T-Cell IFNγ and IL4 Responses

Five mice from each group were used for collection of spleen cells on days 45 and 101. ELISPOT assays for IFNγ and IL4 were performed on spleen cells for each group. T-cell IFNγ responses were measured as described previously [23,24]. Briefly, frozen spleen cells were thawed, washed in RPMI 1640 media supplemented with 10% fetal bovine serum (Hyclone, Logan, Utah) and 1% penicillin-streptomycin (Corning), and placed in a 37 °C 5% CO_2_ incubator overnight for viability recovery. Cells were then plated in ELISPOT plates (MAIPSWU10, Millipore) pre-coated with anti-IFNγ monoclonal antibody or anti-IL4 (kit #3321M-2H or 3421M-2A, Mabtech AB, Stockholm, Sweden) for mock- and antigen-stimulated cultures (1 × 10^5^ cells per well) and for positive control wells (3.3 × 10^4^ cells per well). CPrME peptide pools comprised of 15–20-mer peptides overlapping by 5–11 amino acids from prM (synthesized by Gen-Script USA Inc., Piscataway, NJ, USA) and C/E proteins (BEI Resources) corresponding to DENV2 were used as antigens to stimulate spleen cells at a final concentration of 1 µg/mL per peptide. Cultures treated with diluted solvent (dimethyl sulfoxide) only were used as negative controls (mock control). Positive controls were treated with the mitogen concanavalin A (Sigma-Aldrich) at 2 µg/mL final concentration. Depending on the number of cells recovered after thawing, samples were run in duplicate or triplicate. After a 24-h incubation, the plates were washed and ELISPOT was developed using a mouse IFNγ ELISPOT kit (#3321M-2H, Mabtech AB, Stockholm, Sweden) or mouse IL4 ELISPOT kit (Mabtech #3311-2A) according to the manufacturer’s instructions. The spots were counted on an automated spot counter (AID ELISPOT Reader, Autoimmun Diagnostika GmbH, Straßberg, Germany). The spots were then normalized based on input cells per well and presented as spot forming units (SFUs) per 10^6^ cells. Data were presented as antigen-specific SFUs by subtracting antigen-stimulated SFUs against mock SFUs. Group means between responses from mock and CPrME-stimulated cells were compared using Mann–Whitney unpaired 2-tailed tests. Group means between adjuvants were compared using Kruskal–Wallis tests.

### 2.7. ELISA to Measure the Mouse Immunoglobulin Isotypes

Individual serum samples from each group on day 101 were tested for the presence of different anti-DENV2 IgG isotypes using an Ig Isotyping Mouse Uncoated ELISA kit (ThermoFisher, Waltham, MA, USA). Microplates were coated for 16 h at 4 °C with dengue antigen or negative (uninfected) control antigen (both were derived from Vero cell culture) diluted in phosphate-buffered saline (PBS). Microplates were then blocked with 5% skim milk in PBS for 30 min at room temperature. Mouse sera diluted 1:100 in PBS with 5% skim milk were then added to the plate, incubated for 1 h at 37 °C, and washed six times with 0.1% Tween-20 in PBS using a microplate washer (BioTek, Winooski, VT, USA). Purified rat anti-mouse Ig monoclonal antibodies (IgG1, IgG2a, IgG2b, and IgG3) diluted 1:250 in PBS with skim milk were added to the plate and incubated for 1 h at 37 °C. The plates were washed, and peroxidase-conjugated goat anti-rat IgG (ThermalFisher, Waltham, MA, USA) at 1:10,000 dilution was added. After 1 h of incubation at 37 °C, the plates were washed and tetramethylbenzidine (TMB) substrate solution was added and incubated at room temperature. One molar of formic acid was added after 10 min to stop the reaction. Optical density at 450 nm (OD_450_) was measured using a plate reader (Molecular Devices, Sunnyvale, CA, USA). The OD_450_ value for each mouse serum was calculated by subtracting the OD_450_ value for negative control antigen from the OD_450_ value for dengue antigen. The cutoff value was set as the mean of five PBS group controls plus three standard deviations (SDs). Groups between IgG2a and other IgG isotypes were compared using One-Way ANOVA with Dunnett test.

## 3. Results

### 3.1. Characterization of DENV2 PsIV Vaccine Antigen

**Figure 1:**
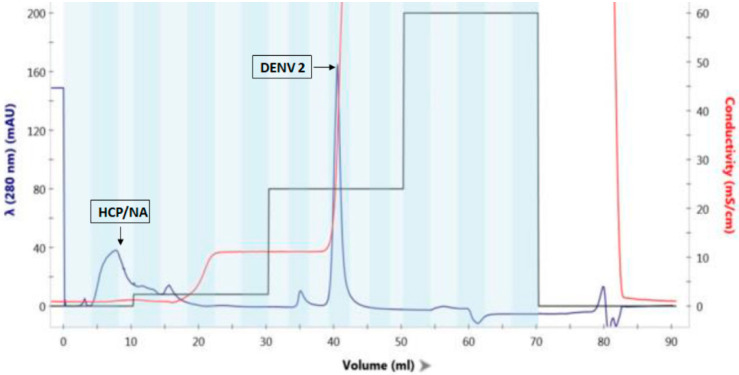
A typical chromatogram illustrating the initial DENV2 purification by Capto DeVirS chromatography resin. The majority of the host cell proteins (HCPs) are removed in the flow through fractions.

**Figure 2:**
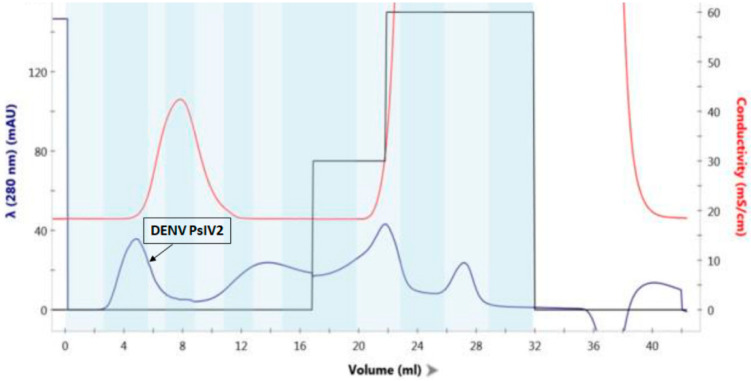
A typical chromatogram illustrating further purification of DENV PsIV2 using the Capto Core 700 column. Dengue virus particles are eluted in the flow-through fractions.

Highly purified DENV2 PsIV was prepared by AMT/UVA inactivation of Capto DeVirS column purified DENV2 followed by a final purification using Capto Core 700 column, as described previously [14]. Briefly, cell culture supernatant containing DENV2 was clarified and desalted using a 50 KDa MWCO filter and then purified on a DeVirS column. Figure 1 illustrates the typical DeVirS column-based initial purification of DENV2. Psoralen derivative AMT was then added to the column-purified DENV2 and irradiated with UVA light to obtain DENV PsIV2, as described in the Materials and Methods section. A final purification of DENV2 PsIV was achieved using a Capto Core 700 column, as illustrated in Figure 2. Fractions containing purified DENV2 PsIV particles were then pooled, mixed with the stabilizer (FTA), filtered through a 0.2 µm filter, and stored at −80 °C. Purified DENV2 PsIV was characterized by Western blot analysis using the anti-flavivirus monoclonal antibody 4G2 to confirm the presence of DENV envelope antigen (Figure S1), and the purity of the DENV2 PsIV vaccine was confirmed by silver staining after gel electrophoresis (Figure S2).

### 3.2. Neutralizing Antibody Responses

Mice were vaccinated on days 0 and 30 for measurement of antibody responses and then on day 90 to help measure T-cell responses. The doses used for both the PIV and PsIV vaccines were chosen with an aim to be suboptimal such that immune response differences between adjuvants could be measured. Sera were collected on days 30, 60, 90, and 101 and used to determine neutralizing antibodies by the MN assay. 

**Figure 3:**
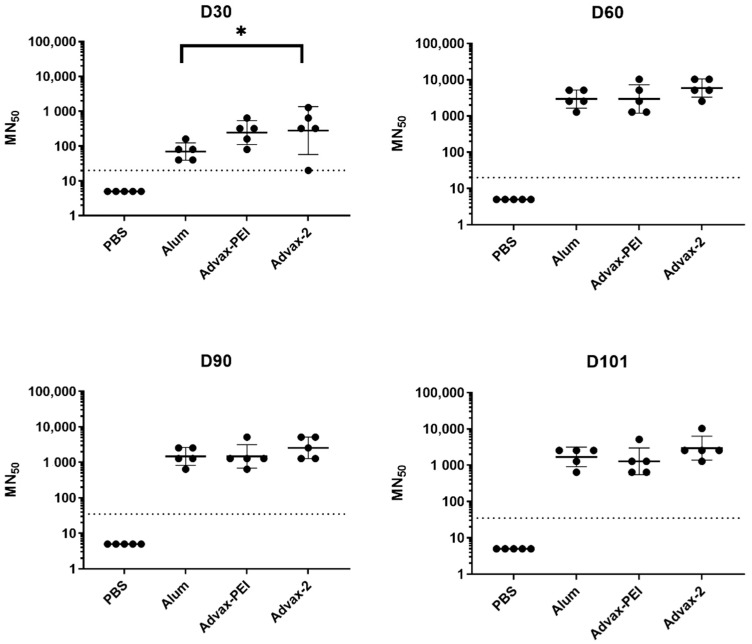
Immunogenicity of monovalent dengue PIV vaccine in mice on days 30, 60, 90, and 101. Each dot represents the DENV-2 MN_50_ titer for an individual mouse. The geometric mean titer for each group is represented by a solid horizontal line. The dotted horizontal line represents the limit of detection for the assay. * indicates significantly (*p* < 0.05) different titers compared to the alum group.

For the PIV groups, after one vaccination, the geometric mean MN_50_ titers in the Advax-PEI and Advax-2 groups were higher than that in the alum group, with the Advax-2 group titers being significantly higher than the alum group titers (*p* = 0.04). Peak geometric mean MN_50_ titers for the PIV groups were observed on day 60 and were highest in the Advax-2 group. Geometric mean MN_50_ titers also look the same for alum and Advax-PEI on days 60 and 90. The only significant difference between the novel adjuvant groups and the alum group was observed on day 30 (Figure 3). 

**Figure 4:**
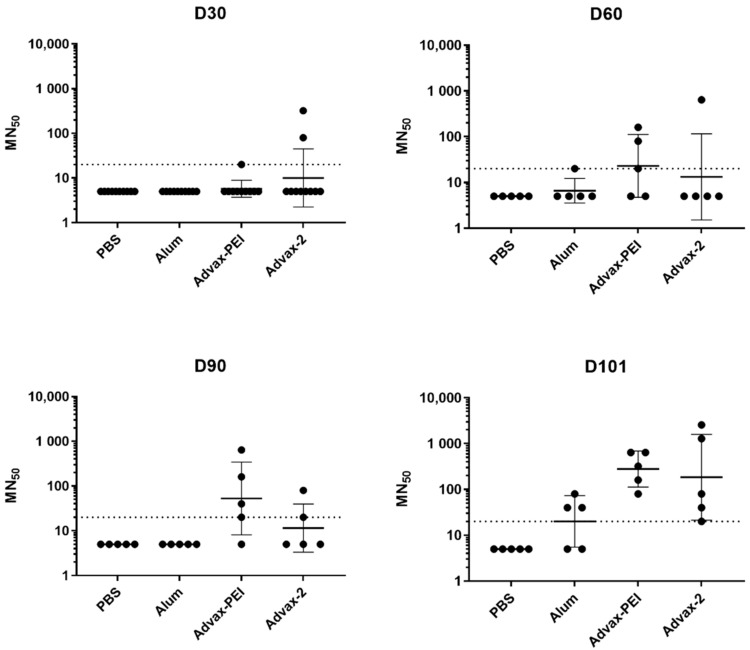
Immunogenicity of monovalent dengue PsIV vaccine in mice on days 30, 60, 90, and 101. Each dot represents the DENV-2 MN_50_ titer for an individual mouse. The geometric mean titer for each group is represented by a solid horizontal line. The dotted horizontal line represents the limit of detection for the assay.

In the PsIV groups, both the Advax-PEI and Advax-2 groups had higher MN_50_ titers than the PBS control group on day 101, and trended higher than the alum control group on day 101, although this difference did not reach statistical significance (Figure 4). 

**Figure 5:**
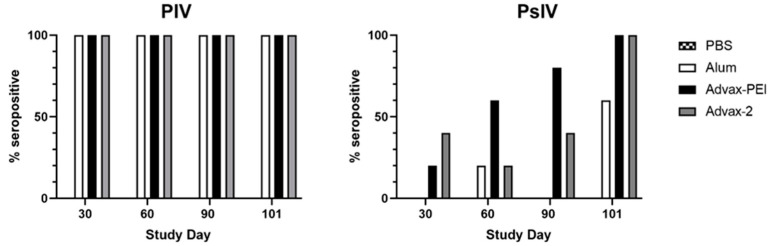
Percentage of mice in each group with an MN_50_ titer ≥ 1:20 by study day. N = 5 for each group.

Results were also analyzed by the frequency of animals in each group that had a positive MN_50_ titer at each time point, whereby positivity was defined as an MN_50_ titer ≥ 1:20. All animals in all groups seroconverted after the first PIV dose. Although, on average, MN_50_ titers waned by about half between days 60 and 90, 100% of animals in all PIV groups remained seropositive on day 101 (Figure 5). In the Advax-2 group, 2/5 (40%) mice had positive MN_50_ titers on day 30 (after the first PsIV dose) compared to 1/5 (20%) in the Advax-PEI group and none in the alum group. By day 60 (after the second PsIV dose), 3/5 (60%) of mice in the Advax-PEI group had become seropositive, and this increased to 4/5 mice (80%) on day 90 and 5/5 (100%) on day 101. In the Advax-2 group, there was more variability with 2/5 (40%) seropositive on day 90 and 5/5 (100%) seropositive on day 101. For the alum group, only 1/5 (20%) of the mice were seropositive after the second dose and only 3/5 (60%) were seropositive after the third dose (Figure 5).

### 3.3. ELISPOT Assays for Measurement of Cell-Mediated Immunogenicity 

**Figure 6:**
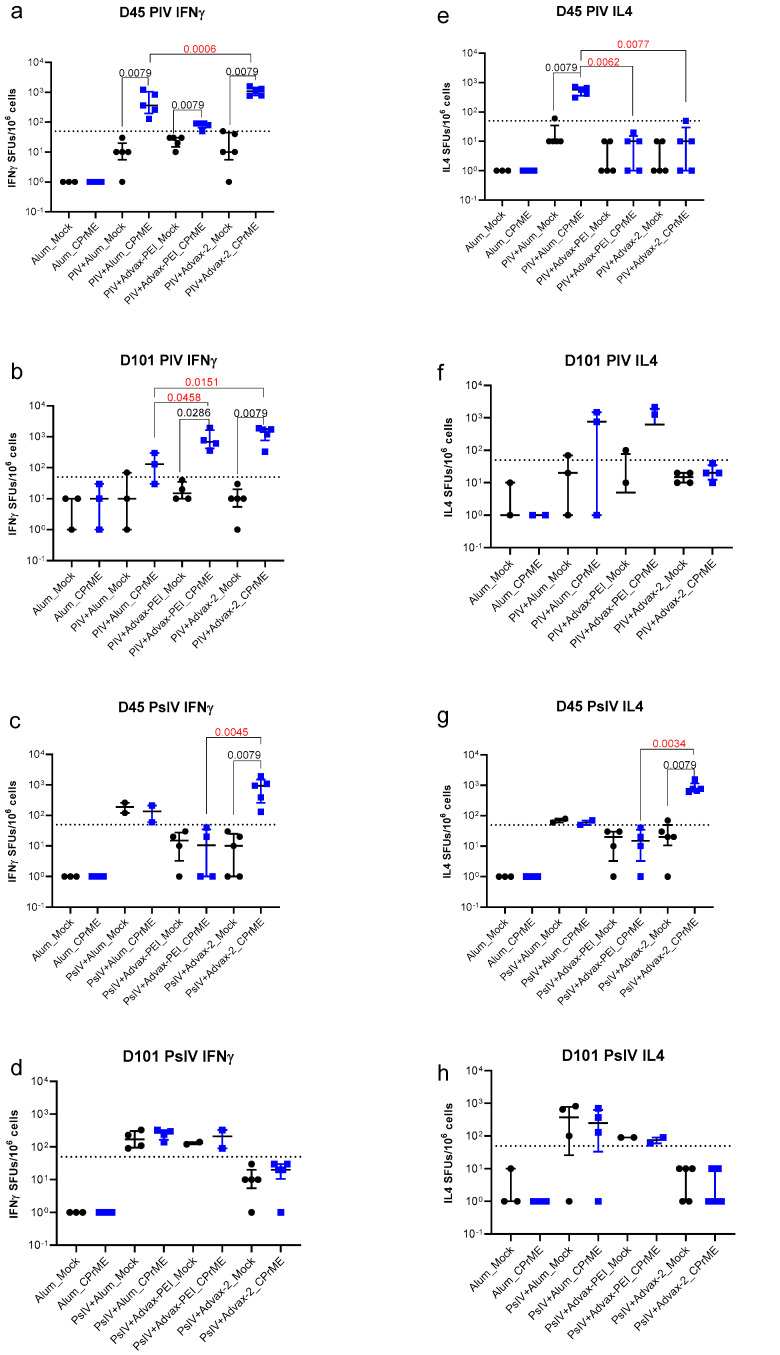
IFNγ and IL4 T-cell responses on days 45 and 101. Antigen specific spot forming units (SFUs) of IFNγ (**a**–**d**) or IL4 (**e**–**h**) producing cells per 10^6^ spleen cells by group and day. Group means between responses from mock and CPrME peptide pool-stimulated cells were compared using Mann–Whitney unpaired 2-tailed tests. Group means between adjuvants were compared using Kruskal–Wallis tests. Only statistically significant differences between groups are marked, and *p*-values were shown. The *p*-values marked in red indicate the differences between different adjuvant groups.

Five mice from each group were used for the collection of spleen cells on days 45 and 101. For some groups only a subset of mice was analyzed due to cell viability issues. ELISPOT assays for IFNγ and IL4 were performed on spleen cells from mice in each group. A threshold of 50 SFUs/10^6^ cells was used to score a positive ELISPOT response. Significant IL4 responses compared to background were only seen on day 45 and were restricted to just the alum-adjuvanted PIV and Advax-2 adjuvanted PsIV groups (Figure 6). Significant IFNγ responses were seen in all the adjuvanted PIV groups at both days 45 and day 101, but with the highest overall responses in the Advax-2 adjuvanted group. For PsIV groups, significant IFNγ responses were only seen in the Advax-2 group. 

The IFNγ to IL4 ratios are shown in Figure S3. For PIV there was a high IFNγ to IL4 ratio for Advax-PEI (day 45) and Advax-2 (both day 45 and day 101) and a low ratio for Alum (day 101), which is consistent with alum having a strong Th2 bias whereas by comparison Advax-2 induced a mixed Th1 and Th2 response. 

### 3.4. Immunoglobulin G Isotypes

**Figure 7:**
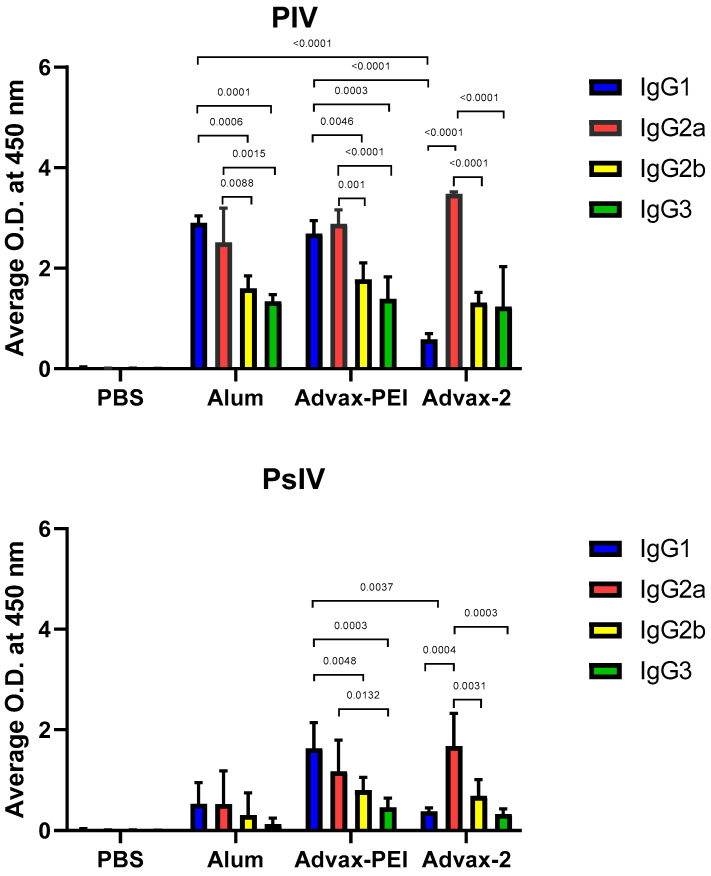
Immunoglobulin G (IgG) isotyping of PIV and PsIV vaccines in mouse serum samples on day 101. Specific IgGs against dengue antigen were tested. There were five mice in each group. The average OD at 450 nm of each group is represented by the height of the column, and the standard deviation is shown as an error bar. IgG1 between different adjuvants and groups between IgG2a and other IgG isotypes were compared using One-Way ANOVA with Dunnett test. Only statistically significant difference between groups were marked, and *p*-values were shown.

In order to determine the distribution of IgG isotypes in vaccinated mice, serum from individual mice collected on day 101 was diluted and tested with an IgG isotyping kit. For mice that received the PIV vaccine with either alum or Advax-PEI, the levels of IgG1 and IgG2a were significantly higher than the levels of IgG2b and IgG3. Additionally, the IgG1 levels of the mice receiving the PIV with either alum or Advax-PEI were much higher than the mice with Advax-2. In mice that received either the PIV or PsIV vaccine with Advax-2, IgG2a was the predominant isotype detected, with IgG1, IgG2b, and IgG3 detected at significantly lower levels (Figure 7). 

## 4. Discussion

In this study, we tested two novel adjuvants to see if they could enhance the immunogenicity of two different types of inactivated dengue vaccines in mice, one where formalin was used for inactivation with an effect on both proteins and nucleic acids and the other, psoralen, which inactivates at the nucleic acid level. Our goal was to use the data from this mouse study to select the most immunogenic adjuvant for each inactivated vaccine candidate to move forward to a NHP study. Neutralizing antibodies were the primary criteria used for down selection, while T-cell responses were also considered.

Previous mouse studies with the dengue PIV vaccine using alum as the adjuvant demonstrated that while it could elicit high-titer virus neutralizing antibodies, it was not effective at eliciting cell-mediated immune responses, and the neutralizing antibody titers waned quickly over time [22]. Therefore, we tested two novel adjuvants (Advax-PEI and Advax-2) to see if they could further boost the antibody titer and durability of the dengue PIV response. For the PIV groups, MN_50_ titers were the same across the board, with the exception at day 30 where Advax-2 was significantly higher than alum after the first vaccine dose. There was a high rate of seropositivity in all groups, which was maintained out to day 101, even though some waning of the titers was observed after their peak on day 60. While we were aiming to use a suboptimal dose of PIV, it is possible that the dose used was too high and the neutralizing antibody responses were saturated after the second dose. This phenomenon could explain why large differences in MN titers between the adjuvant groups were only observed after the first dose. 

For the PsIV vaccine, we were also aiming for a suboptimal dose to better see the effect of the adjuvants. Unfortunately, the dose used was likely too low based on the lower geometric mean of MN_50_ titers generated by the PsIV vaccine. Although the PsIV dose used (10^5^ particles per dose) turned out to be too low, it provided an opportunity to assess the dose-sparing effects of the adjuvants with this antigen. Even at this low dose, only PsIV formulated with Advax-PEI and Advax-2 induced detectable neutralizing antibody titers in some animals at day 90 and in 100% of animals at day 101. By contrast, 0 of 5 mice in the alum group had detectable antibodies at day 90, and only 3 of 5 mice at day 101. Notably, on day 101 of the current study, all mice in the Advax-PEI and Advax-2 groups remained seropositive. 

In a previous mouse study, both the monovalent and tetravalent DENV PsIVs elicited good neutralizing antibody responses that persisted out to eight weeks after administration of the second dose at the higher dose tested (10^6^ particles per dose), while a poor antibody response was observed at the lower dose tested (10^4^ particles per dose) [14]. In the previous study, a lower dose (10^4^ particles) of alum-adjuvanted DENV2 PsIV elicited MN_50_ titers of 600 at day 60 (30 days after second immunization) [12]. This is a 10-fold lower dose than what was used in this study, yet higher MN_50_ titers were observed. It was surprising that no detectable neutralization was observed at the same time point (day 60) for alum-adjuvanted DENV2 PsIV in this study. However, in this study we used intramuscular immunization, whereas the previous one used intradermal immunization. We used intramuscular immunization in this study because it was recommended by our coauthors at WRAIR. Only five mice per group were used for serology because we sacrificed a further five mice per group to determine T-cell responses. Poor immune response at the lower dose tested in this study may be due to stability issues of the purified vaccine over time. Although we have added stabilizers to the purified vaccines prior to freezing them, a thorough stability study of the highly-purified DENV PsIVs over long periods of storage and number of freeze-thaw cycles has not been performed yet. In our previous study, we utilized freshly prepared DENV PsIV vaccines (within 1 week of preparation). Therefore, a thorough stability study of highly purified DENV PsIV vaccines using various excipients as well as under different storage conditions over a long period of storage and number of freeze-thaw cycles is warranted. 

A subset of mice from each group was used for collection of spleen cells for T-cell ELISPOT assays for IFNγ and IL4 on days 45 and 101. Advax-2 typically induces IFNγ (a marker of Th1 immunity) and either stimulates or suppresses IL4 (a marker of Th2 immunity) [25]. For the PIV vaccine, only IFNγ ELISPOT were seen in the Advax-2 adjuvanted PIV mice, indicating a Th1 dominant response. By contrast, alum-adjuvanted PIV induced an IL4 response, consistent with alum’s known Th2 bias. However, for the PsIV vaccine, Advax-2-vaccinated mice showed both IFNγ and IL4 ELISPOT responses. Notably, when formulated with either PIV or PsIV vaccine, Advax-2 induced an IgG2a dominant isotype response, with IgG2a isotype switching being a marker of a Th1 response [26]. Hence, Advax-2-adjuvanted PIV or PsIV vaccines elicited high neutralizing antibody titers and Th1 dominant T-cell responses, as indicated by increased IFNγ and IgG2a production. This all supports Advax-2′s suitability for use as an adjuvant in dengue vaccine development. While the mechanism of Advax adjuvants enhancing Th1 immune responses remains to be fully elucidated, labelled-Advax adjuvant particles are known to be avidly phagocytosed by macrophages and dendritic cells, resulting in upregulation of MHC and costimulatory molecules on these cells [27]. In response, there is a major expansion of antigen-specific IFN-γ secreting CD4 and CD8 T-cells consistent with Advax adjuvant, providing a strong Th1 stimulus, although memory T-cells secreting Th2 cytokines are also expanded [28]. Advax has been described as an adjuvant of adjuvants, which explains its ability to enhance the activity of co-administered traditional adjuvants such as TLR9 agonist CpG oligonucleotides, the second component in Advax-2 [29]. A CMV vaccine study of Advax adjuvants in monkeys showed Advax adjuvants induced strong antibody and memory CD4 and CD8 T-cells in the absence of activation of inflammatory gene pathways [30]. Hence, Advax adjuvants utilize non-inflammatory mechanisms to enhance memory T-cell generation and induction of affinity-matured plasmablasts and memory B cells [31]. 

The limitations of the current study were the relatively small number of mice in each group, which limited statistical comparisons. In addition, the PsIV dose was too low, whereas the dose of PIV was too high, limiting our ability to make robust comparisons. The study also lacked challenge data (usually performed in the NHPs, but not in mice) to see how the observed immune responses might translate into protection. Nevertheless, despite these limitations, this study provides useful information on the effects of the novel adjuvants with the two inactivated dengue virus vaccine platforms, allowing selection of a lead vaccine-adjuvant formulation for further development. Historically, non-human primates such as rhesus or cynomolgus macaques have been used to measure dengue vaccine efficacy, which is our next step. Our long-term goal is to develop a rapid acting, safe and well tolerated prophylactic vaccine platform to protect military and civilian personnel against dengue fever. Ideally, the vaccine should induce protective immunity with a short dosing schedule and should provide protection in individuals who have never been exposed to the dengue virus before. In addition to dengue virus, other medically important flaviviruses can cause severe disease. This inactivated vaccine-adjuvant platform could be expandable and allow the additional flavivirus antigens to further broaden flavivirus coverage provided by a single vaccine platform. GlaxoSmithKline plc (GSK) deprioritized development of the formalin-inactivated dengue PIV vaccine with our collaborators at WRAIR in 2018 because of lack of a durable immune response and ADE concerns; thus, one of the risk mitigations was to explore new adjuvants to circumvent these issues and allow the dengue PIV and PsIV vaccine development to continue. Because psoralen-inactivation of dengue virus occurs at the nucleic acid level, leaving the structural proteins intact, we believe it is an ideal candidate for formulating a tetravalent PsIV DENV vaccine for evaluation in NHP challenge mode. An effective tetravalent DENV vaccine consisting of an optimized ratio of different monovalent PsIV antigens should elicit uniform neutralizing antibody responses against all four serotypes to reduce ADE concerns. In addition, Advax-2 adjuvant itself may help to reduce the risk of ADE based on recent data where it modified the function of JEV vaccine-induced antibodies such that they no longer enhanced DENV1 or DENV2 virus uptake in an in vitro dengue ADE reporter cell assay [20], although any ability of our adjuvanted dengue vaccine to reduce potential ADE effects remains to be investigated.

## 5. Conclusions

Successful dengue inactivated vaccines are likely to be highly dependent on identification of suitable adjuvants. We tested two novel adjuvants against alum for their ability to increase the immunogenicity of inactivated dengue antigens. For the PIV groups, after one dose, MN_50_ titers were higher in the Advax adjuvant groups compared to the alum control, while titers were comparable between the adjuvant groups after the second dose. For the PsIV groups, both Advax adjuvants induced higher MN_50_ titers than the alum control after the second dose. Overall, Advax-2 responses were at least as good if not fractionally superior to Advax-PEI, with the biggest distinction being that Advax-2 already has extensive human clinical data and is included in a now-licensed COVID-19 vaccine; therefore, the Advax-2 formulation will be pursued for further development of the dengue vaccine. The NIH is supporting development of Advax adjuvants, including Advax-2, across a range of indications including influenza, COVID-19, and HIV vaccines, and Advax-2 adjuvant is already available as a GMP product and has been shown to be safe and highly effective in humans, thereby providing confidence of availability and regulatory acceptance for use in an inactivated dengue vaccine. The lead candidate vaccine formulations are currently being tested in NHP studies, with follow-on studies planned to be conducted to test tetravalent dengue vaccine formulations in investigational new drug (IND)-enabling safety studies to facilitate a future Phase I clinical trial. 

## Figures and Tables

**Table 1:** Vaccination groups for immunogenicity study in mice. Two doses of the vaccine-adjuvant formulations were administered to all animals intramuscularly on day 0 and day 30.

Group	Vaccine-Adjuvant Formation	Number of Mice
1	PIVD2 + Alum	10
2	PsIVD2 + Alum	10
3	PIVD2 + Advax-PEI	10
4	PIVD2 + Adxax-2	10
5	PsIVD2 + Advax-PEI	10
6	PsIVD2 + Adxax-2	10
7	Alum	10

PIVD2 = Purified-inactivated vaccine DENV2; PsIVD2 = Psoralen-inactivated DENV2; Advax-PEI = Delta inulin; Advax-2 = Delta inulin + CpG55.2 oligonucleotide.

**Table 2:** Schedule of procedures: Immunization regimen and sample harvest.

Procedure	Day of Study					
	0	30	45	60	90	101
Vaccination (*n* = 10)	X	X			X	
Bleeding (*n* = 10)	X	X		X	X	X
Spleen harvest (*n* = 5)			X			X

## Supplementary Materials

The following supporting information can be downloaded at: https://www.mdpi.com/article/10.3390/microorganisms10051034/s1. Figure S1. Western blot analysis of Capto Core 700 column fractions run on Novex 4–12% Tris-Glycine SDS gel. Primary antibody is 4G2 anti-flavivirus monoclonal antibody specific for envelope protein (as indicated by arrow). Capto Core fractions 2 to 4 containing purified DENV-2 PsIV were pooled together and stored at −80 °C (after adding the stabilizers). Figure S2. Silver Stain of Capto Core 700 column fractions run on Novex 4−12% Tris-Gylcine native gel. Fractions 2 to 4 contain highly purified DENV-2 PsIV. Figure S3. Log 2 ratio of IFNγ and IL4 for data in Figure 6 is shown for responses to D2CPrME. Ratio for responses to Mock is not shown.
