# Supplementary material for: Enhanced Immunogenicity of Inactivated Dengue Vaccines by Novel Polysaccharide-Based Adjuvants in Mice
Source: Microorganisms. 2022 May 16;10(5):1034. doi: 10.3390/microorganisms10051034 (PMC9146336; doi:10.3390/microorganisms10051034)
Supplement: Supplementary file 1 [file microorganisms-10-01034-s001.zip › Figure S2.pdf]

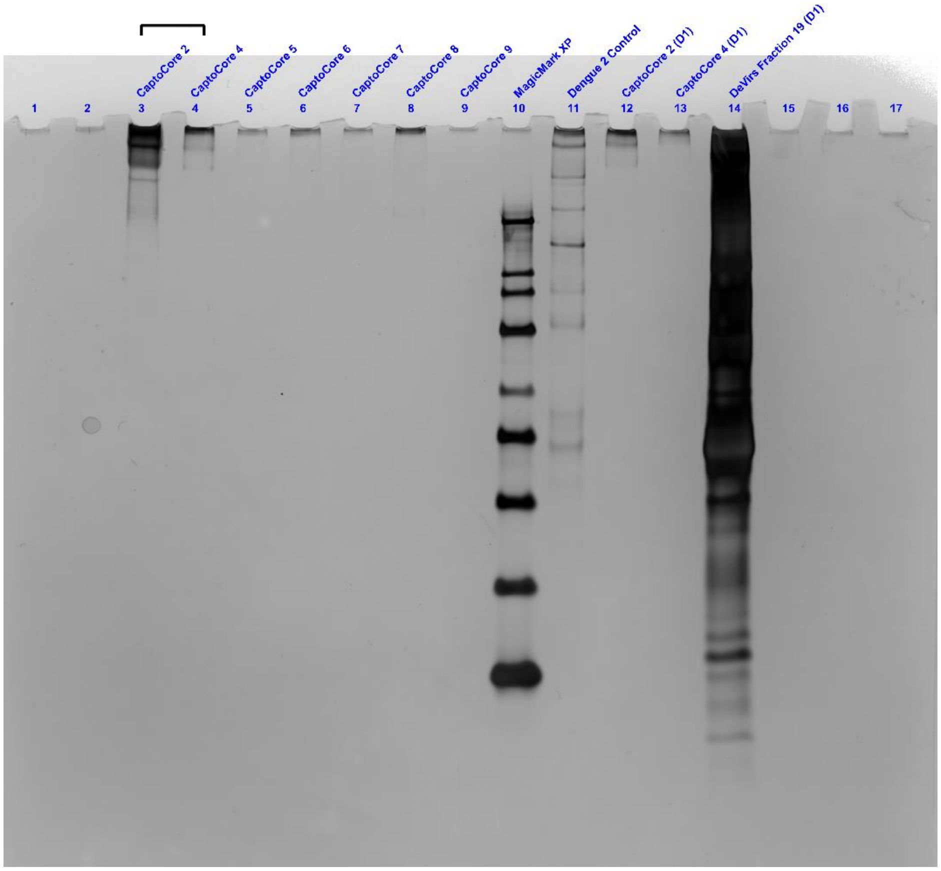

Figure S2. Silver Stain of Capto Core 700 column fractions run on Novex 4-12% Tris-Gylcine native gel. Fractions 2 to 4 contain highly purified DENV-2 PsIV.
