# Supplementary material for: Enhanced Immunogenicity of Inactivated Dengue Vaccines by Novel Polysaccharide-Based Adjuvants in Mice
Source: Microorganisms. 2022 May 16;10(5):1034. doi: 10.3390/microorganisms10051034 (PMC9146336; doi:10.3390/microorganisms10051034)
Supplement: Supplementary file 1 [file microorganisms-10-01034-s001.zip › Figure S3.pdf]

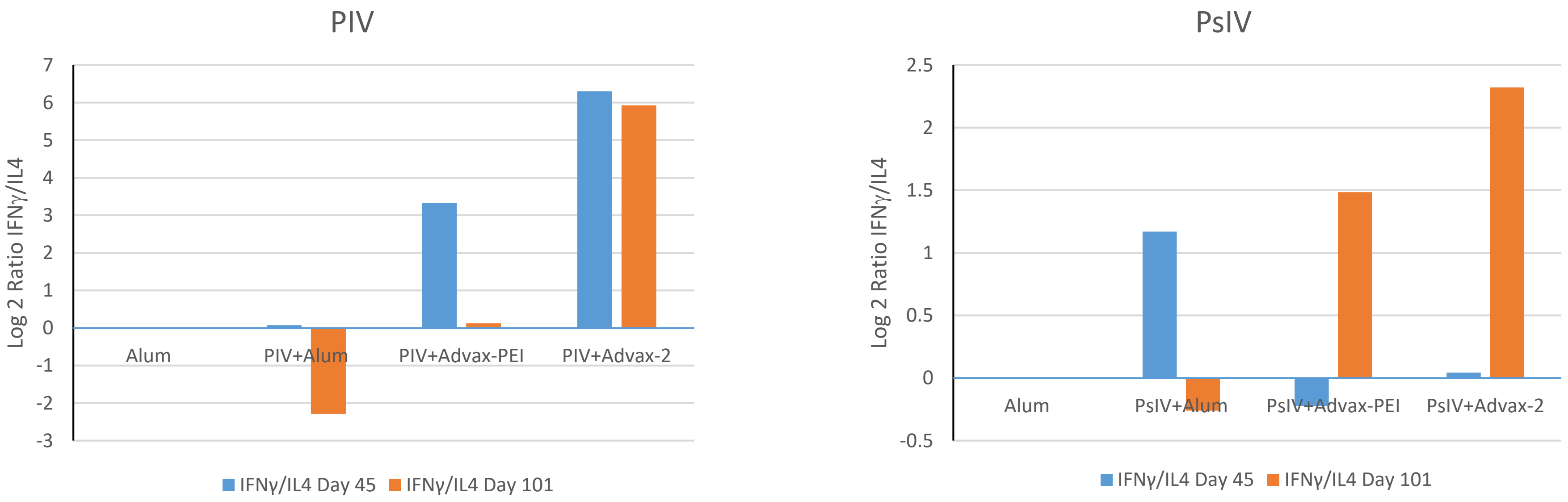

Figure S3. Log 2 ratio of IFNγ and IL4 for data in Figure 6 is shown for responses to D2CPrME. Ratio for responses to Mock is not shown.
