# Supplementary material for: Enhanced Immunogenicity of Inactivated Dengue Vaccines by Novel Polysaccharide-Based Adjuvants in Mice
Source: Microorganisms. 2022 May 16;10(5):1034. doi: 10.3390/microorganisms10051034 (PMC9146336; doi:10.3390/microorganisms10051034)
Supplement: Supplementary file 1 [file microorganisms-10-01034-s001.zip › Figure S1.pdf]

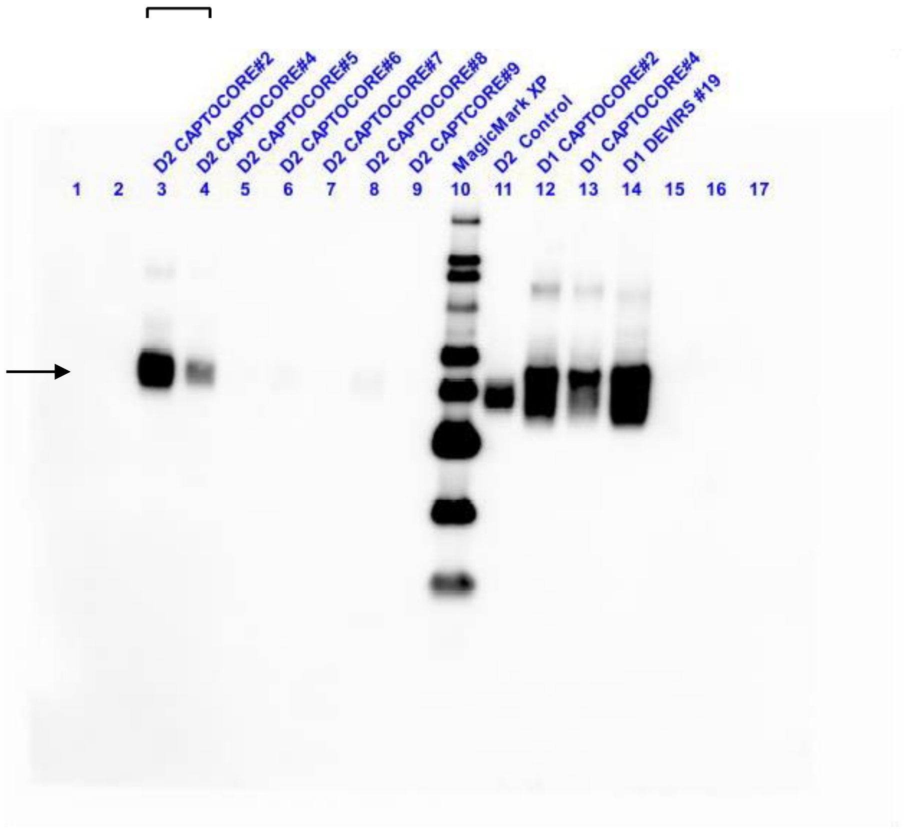

Figure S1. Western blot analysis of Capto Core 700 column fractions run on Novex 4-12% Tris-Glycine SDS gel. Primary antibody is 4G2 anti-flavivirus monoclonal antibody specific for envelope protein (as indicated by arrow). Capto Core fractions 2 to 4 containing purified DENV-2 PsIV were pooled together and stored at -80 °C (after adding the stabilizers).
